# Multi-Q 2 software facilitates isobaric labeling quantitation analysis with improved accuracy and coverage

**DOI:** 10.1038/s41598-021-81740-4

**Published:** 2021-01-26

**Authors:** Ching-Tai Chen, Jen-Hung Wang, Cheng-Wei Cheng, Wei-Che Hsu, Chu-Ling Ko, Wai-Kok Choong, Ting-Yi Sung

**Affiliations:** 1 Institute of Information Science, Academia Sinica, 128 Academia Road, Section 2, Nankang, Taipei, 115 Taiwan; 2 Bioinformatics Program, Taiwan International Graduate Program, Academia Sinica, Taipei, 115 Taiwan; 3 Institute of Biomedical Informatics, National Yang-Ming University, Taipei, 112 Taiwan; 4 Genomics Research Center, Academia Sinica, Taipei, 115 Taiwan; 5 Department of Computer Science and Engineering, University of California San Diego, La Jolla, CA 92093 USA

**Keywords:** Bioinformatics, Proteomics, Proteome informatics, Software

## Abstract

Mass spectrometry-based proteomics using isobaric labeling for multiplex quantitation has become a popular approach for proteomic studies. We present Multi-Q 2, an isobaric-labeling quantitation tool which can yield the largest quantitation coverage and improved quantitation accuracy compared to three state-of-the-art methods. Multi-Q 2 supports identification results from several popular proteomic data analysis platforms for quantitation, offering up to 12% improvement in quantitation coverage for accepting identification results from multiple search engines when compared with MaxQuant and PatternLab. It is equipped with various quantitation algorithms, including a ratio compression correction algorithm, and results in up to 336 algorithmic combinations. Systematic evaluation shows different algorithmic combinations have different strengths and are suitable for different situations. We also demonstrate that the flexibility of Multi-Q 2 in customizing algorithmic combination can lead to improved quantitation accuracy over existing tools. Moreover, the use of complementary algorithmic combinations can be an effective strategy to enhance sensitivity when searching for biomarkers from differentially expressed proteins in proteomic experiments. Multi-Q 2 provides interactive graphical interfaces to process quantitation and to display ratios at protein, peptide, and spectrum levels. It also supports a heatmap module, enabling users to cluster proteins based on their abundance ratios and to visualize the clustering results. Multi-Q 2 executable files, sample data sets, and user manual are freely available at http://ms.iis.sinica.edu.tw/COmics/Software_Multi-Q2.html.

## Introduction

Mass spectrometry-based proteomics has become a dominating approach for identification and quantitation of proteins from complex biological samples^1^. Among several quantitation techniques, isobaric labeling-based quantitative proteomics has gained increasing attention because of its multiplexed capability, allowing for absolute and relative protein quantitation in multiple samples within a single run^2,3^. For example, a number of research groups in CPTAC (Clinical Proteomic Tumor Analysis Consortium)^4,5^ use isobaric labeling experiments, such as iTRAQ (isobaric Tags for Relative and Absolute Quantitation) or TMT (Tandem Mass Tag), to quantify thousands of proteins for cancer tissue samples from hundreds of patients.

Two major objectives of bioinformatics analysis of isobaric labeling experiments are enlarging quantitation coverage (i.e., the number of quantified proteins) and improving quantitation accuracy. Enlarging quantitation coverage increases the likelihood of finding important biomarkers. To date, a number of isobaric-labeling quantitation software tools support identification results from only a single database search engine for quantitation. For example, IsobariQ^6^ and jTraqX^7^ exclusively support Mascot; MaxQuant^8^, PatternLab^9^, and OCAP^10^ exclusively support Andromeda^11^, Comet^12^, and X!Tandem^13^, respectively. The quantitation coverage for these tools could be limited because it has been reported that combining multiple search engines can markedly increase the numbers of identified peptides and proteins^14^.

For proteomics experiments in search of biomarkers, improving quantitation accuracy decreases the possibilities of false positives (misclassification of proteins with insignificant fold changes as differentially expressed) and false negatives (misclassification of differentially expressed proteins as having insignificant fold changes). Isobaric-labeling quantitation workflow usually consists of three major components: calculation of ratio of protein abundances in two samples (termed protein ratio), normalization, and reducing the effect of ratio compression^15,16^ on quantitation accuracy. Protein ratio calculation takes a set of identified PSMs as input to infer protein ratios. Common approaches include the use of mean^17^, median^7,17,18^, weighted average^19–21^, and 20% trimmed mean^16,17^ of the ratios at the spectrum level (PSM ratios) to determine protein ratios. Another common approach aggregates or sums the reporter ion intensities (usually after normalization) across different PSMs, and then infers protein ratios based on the aggregate values^22^. Instead of deriving protein ratios directly from PSMs, some other methods adopt a hierarchical two-stage procedure, i.e., PSM ratios to infer peptide ratios (stage one), and peptide ratios to infer protein ratios (stage two). Commonly used two-stage quantitation algorithms adopt weighted average^23^ and median^6,24^ for both stages.

Normalization is usually applied at reporter ion level, in which reporter ion intensity of each channel is normalized according to its mean^17^, median^6,17,19,20^, or summation^6,17^ of intensities across all the spectra. Normalization can also be applied at the level of peptide or protein ratio based on the assumption that the median of log transformed peptide or protein ratios is zero^6,7,23^. Variance stabilizing normalization is also proposed to address the issue of heterogeneity of variance among peaks of varying intensities^6,16,17,25^. Ratio compression arises from the presence of coeluting peptides within a precursor isolation window, resulting in systematic underestimation of actual protein and peptide abundance variations^16,26–29^. Using triple-stage mass spectrometry (MS3)^30,31^ or dual isolation width acquisition^32^ can mitigate the negative impact of these interfering signals, but they require modifications in the experimental settings or the instrument control code and are not applicable to the majority of existing isobaric labeling data sets. On the other hand, an algorithm that corrects compressed ratios based on calculated signal-to-interference ratios has been proposed^33^, offering an attractive solution to ratio compression.

Quantitation results depend on the combination of algorithms for protein ratio calculation, normalization, and correction of ratio compression. In spite of great efforts being made to devise the above-mentioned algorithms, there is a lack of systematic evaluation of all the algorithmic combinations with various scoring functions. In light of the situation, we develop a standalone and installation-free software tool, Multi-Q 2, which is equipped with six protein ratio calculation algorithms, four peptide ratio calculation algorithms, three normalization algorithms, and the ratio compression correction (RCC) algorithm. With Multi-Q 2, we generate up to 336 algorithmic combinations and systematically evaluate them with standard data sets using various evaluation measures. Our analyses reveal that different algorithmic combinations have different strengths and are suitable in different scenarios. To be specific, algorithmic combinations that better quantify proteins of larger fold changes tend to sacrifice the accuracy for proteins of smaller fold changes, and vice versa. Thus, it is preferable that a software tool provides the flexibility of customizing quantitation algorithms for different purposes. With proper selection of algorithmic combinations, Multi-Q 2 can achieve better quantitation accuracy than MaxQuant, PatternLab, and Libra^34,35^. We further demonstrate that while searching for differentially expressed proteins, a set of six selected algorithmic combinations produce 70% to 90% common candidate proteins, suggesting that using algorithmic combinations of complementary rationale simultaneously in search of differentially expressed proteins can be a reasonable strategy to increase either sensitivity or precision.

Multi-Q 2 is a Windows-based software tool designed with great flexibility. It supports identification results from Trans-Proteomic Pipeline (TPP)^34,35^, PeptideShaker^36^, and the commercial Proteome Discoverer, offering up to 12% improvement in coverage compared with MaxQuant and PatternLab, both of which are limited to identification results of a single database search engine. Multi-Q 2 provides interactive graphical user interfaces to perform quantitation and to display protein, peptide, PSM ratios as well as raw and processed reporter ion intensities. Moreover, a heatmap module is implemented to cluster proteins based on the calculated ratios using hierarchical clustering, K-means, and fuzzy C-means, facilitating users to perform further investigations.

## Methods and data sets

### Multi-Q 2 for isobaric-labeling quantitation analysis

#### Overview of Multi-Q 2

**Figure 1:**
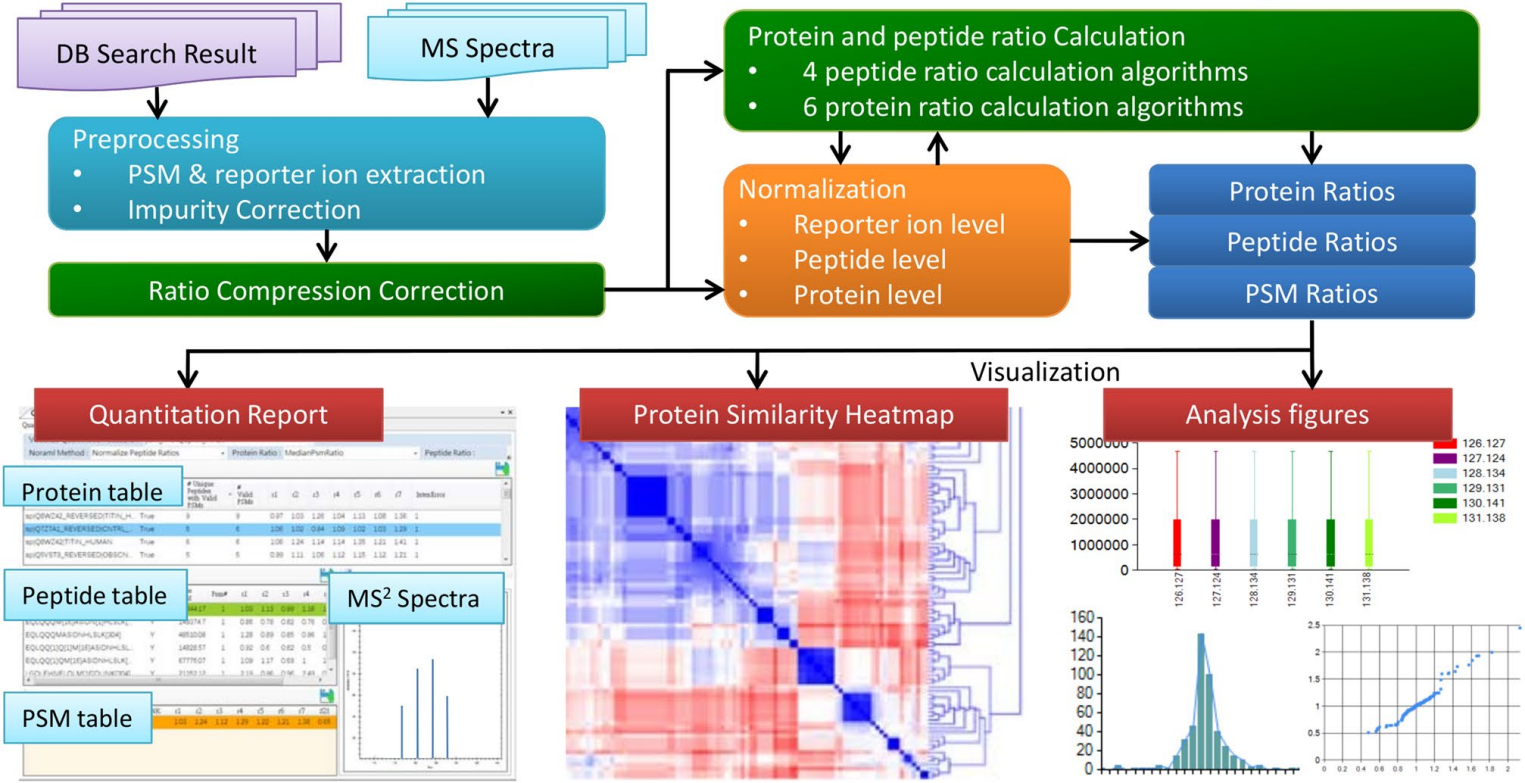
Overview of Multi-Q 2.

As illustrated in Fig. 1, Multi-Q 2 takes database search results and MS spectra files as input, and then performs standard preprocessing steps, including reporter ion extraction and isotope impurity correction. A ratio compression correction (RCC) algorithm is implemented to resolve the isobaric interference problem caused by co-eluting peptide ions in the precursor isolation window. Multi-Q 2 supports various commonly used algorithms to calculate protein and peptide ratios, and normalization algorithms applied at reporter ion, peptide, and protein levels. Quantitation results in terms of protein, peptide, and PSM ratios can be visualized through interactive graphical user interfaces. A heatmap as well as a dendrogram can be generated with unsupervised learning on protein ratios to characterize similarities among proteins.

#### Input format and preprocessing of Multi-Q 2

Multi-Q 2 accepts MS spectra files in mzML or mzXML format. It accepts statistically validated search results from three popular proteomics data analysis systems: (1) pep.xml and prot.xml files from TPP, (2) xls files from PeptideShaker, and (3) pep.xml and prot.xml files from Proteome Discoverer. Statistical validation is usually performed with a target-decoy search strategy which provides estimation of false discovery rate (FDR) for given PSMs and thus can distinguish correct PSMs from incorrect PSMs under a specified FDR^37^. To ensure correct PSMs for further quantitation, Multi-Q 2 takes the spectra satisfying an FDR of 1% at peptide and protein levels by default and extracts their reporter ions. Isotope impurity correction on reporter ions is then performed based on the correction matrices provided by reagents’ manufacturers^23^. Multi-Q 2 includes preset matrices for iTRAQ-4, iTRAQ-8, TMT-6, TMT-8, and TMT-10. User-defined correction matrix is also supported.

#### Ratio compression correction

An algorithmic solution to the ratio compression issue has been proposed in Savitski et al.^33^ and is shown to improve quantitation accuracy without sacrificing quantitation coverage as spectra filtering approach does^33^. This RCC algorithm is implemented in Multi-Q 2. The assumption of the RCC algorithm is that the total percentage of noise from co-eluting interferences in the reporter ions of an MS^2^ scan is consistent with the percentage of noise of co-eluting interferences versus the total ion signals in its precursor isolation window. The authors calculate the signal-to-interference (S2I) measure as the abundance of a precursor and its isotope cluster divided by the sum of all ion signals observed within the isolation window. S2I ranges from zero to one and a smaller value indicates a higher degree of interferences caused by co-fragmented peptide ions. RCC calculates the total amount of noise in reporter ions as the product of 1-S2I and the summation of all the reporter ion intensities, thereby inferring putative noise for each channel. The putative noise is then deducted from the raw reporter ion intensity of the corresponding channel, and the resulting corrected intensity is used for quantitation in Multi-Q 2.

#### Protein and peptide ratio calculation

Multi-Q 2 calculates PSM, peptide and protein ratios between two selected channels in an experiment at PSM, peptide, and protein levels, respectively. Given a PSM, the corrected reporter ion intensities of the two channels in the spectrum are used to calculate the PSM ratio. Multi-Q 2 supports the following four different approaches to calculate peptide ratios from PSMs: (1) LinearRegression: collect paired reporter ion intensities of two selected channels across all PSMs of a peptide, and use the slope of the linear regression model as the peptide ratio; (2) SumPsmIntensity: sum up the reporter ion intensities across all PSMs of a peptide, and use these aggregate values to derive peptide ratios; (3) MedianPsmRatio: use the median of PSM ratios as the peptide ratio; (4) WeightedPsmRatio: use the weighted average of PSM ratios to derive peptide ratios, in which the weight of a PSM ratio is proportional to the summation of its reporter ion intensities.

To calculate protein ratios, the following six algorithms are supported: (1) SumPsmIntensity: sum up the reporter ion intensities across all PSMs assigned to the protein, and use these aggregate values to derive protein ratios; (2) MedianPsmRatio: use the median of the PSM ratios as the protein ratio; (3) WeightedPsmRatio: same as the WeightedPsmRatio approach for peptide ratio calculation except for the use of all the PSMs assigned to the protein; (4) TrimmedMeanPsmRatio: remove 20% of the largest and the smallest PSM ratios, respectively, and calculate the mean of the remaining PSM ratios as the protein ratio; (5) MedianPepRatio: use the median of the calculated peptide ratios as the protein ratio; (6) WeightedPepRatio: use the weighted average of peptide ratios to derive protein ratios, in which the weight of a peptide ratio is defined as the median of the weights of PSM corresponding to the peptide.

#### Normalization

Multi-Q 2 supports three different levels of normalizations. Normalization at the reporter ion level adjusts reporter ion intensities of respective channels so that their median intensities are identical based on the assumption that the sample amounts across different channels remain constant. Based on the assumption that the amounts of most of peptides (proteins) across different channels, e.g., channels corresponding to samples of patients with different states of a disease, remain the same, normalization of peptide (protein) ratios is to multiply the ratios by the reciprocal of the median peptide (protein) ratio so that the normalized peptide (protein) ratios have a median of 1.

### Data sets

Four data sets were obtained from public domain to evaluate various ratio calculation algorithms and normalization approaches of Multi-Q 2. These are (1) Gatto-TMT6^38^, a data set of *Erwinia carotovora* lysate with four standard proteins at varying ratios, (2) Hultin-iTRAQ8^39^, a standard data set of human A549 cell lysate with known abundance ratios at 2:2:1:1:2:2:1:1, (3) NCI-7^40^, a TMT-10 data set from seven cancer cell lines, and (4) Chen-iTRAQ8^41^, a data set of samples from *anaerobic thermophilic eubacterium*. Detailed descriptions of the data sets can be found in Supplementary Text S1.

### Protein and peptide identification of data sets for further quantitation

Gatto-TMT6 and Hultin-iTRAQ8 were processed by nine different database search methods and statistical validation tools in order to investigate the coverage of the identified proteome. The first eight methods used search engines in SearchGUI^42^ for searches, followed by statistical validation using PeptideShaker, where the first seven methods used a single search engine, including MyriMatch^43^, Andromeda, OMSSA^44^, Tide^45^, Comet, X!Tandem and MSGF+^46^, and the eighth method adopted combined searches using Comet, X!Tandem, and MSGF+. The ninth method used Comet and X!Tandem searches in TPP followed by PeptideProphet^47^, iProphet^48^, and Mayu^49^ for statistical validation. NCI-7 was processed by Comet and X!Tandem searches using SearchGUI and statistical validation using PeptideShaker for protein identification. Chen-iTRAQ8 was processed by Comet, X!Tandem, and MSGF+ searches in TPP followed by PeptideProphet, iProphet, and Mayu. All of the operations in TPP were performed via WinProphet^50^, a software system for proteomic pipeline creation and management. All of the database search parameters were identical to the settings reported in their original papers. Identified proteins and peptides were validated with FDR no greater than 1%.

### Evaluation measures for quantitation results

The coverage of ratios at protein level under a deviation of a certain amount from the ideal value has been used to evaluate the accuracy of quantitation results. Previous studies compare the coverage at 10%, 20%, and 30% deviation for different quantitation tools^17,20,21^. To generalize the concept, we devise a measure called *area under the curve of coverage vs deviation* (*AUCCD*), in which a curve is generated on a 2D plane with x-axis denoting deviation of protein ratios, ranging from 0 to 100% to the ideal values, and y-axis denoting coverage of protein ratios within the deviation. A large AUCCD indicates larger coverage of protein ratios within the specific range (0–100% in our design) of deviation and implies higher accuracy of quantitation. We also use two additional measures: *average relative error* (*ARE*), defined as the average of |*x*_*i*_−*y*_*i*_|/*y*_*i*_ over all the protein ratios, and *root mean square error* (*RMSE*), defined as the square root of Σ_*i*_(*x*_*i*_−*y*_*i*_)^2^/*m*, where *x*_*i*_ and *y*_*i*_ denote calculated and ideal ratios at protein level, respectively, and *m* is the total number of ratios at protein level. Particularly for ARE, since one of our data sets, i.e., Gatto-TMT6, contains standard proteins and background proteins, we further distinguish ARE_Std and ARE_Bg to denote the ARE of ratios for all standard proteins and for all background proteins, respectively. AUCCD focuses on the percentage of protein ratios within a specific range of deviation to their ideal values and ignores the ratios far from the ideal values. RMSE, in contrast, produces much larger penalties for protein ratios of the latter case. ARE assigns larger (smaller) tolerance for protein ratios of larger (smaller) ideal values so that ratios at different scales can be considered on the same basis. The three evaluation measures are designed based on different rationale and are used integrally to provide a more comprehensive understanding for quantitation results.

## Results and discussion

### Comparison of identification results from different database search and validation tools

Since quantitation tools accept identification results for further quantitation, we first compared identification results of two standard data sets, Gatto-TMT6 and Hultin-iTRAQ8, from using a single search engine or multiple search engines, followed by validation using different tools. Supplementary Figs. S1 and S2 show the numbers of identified proteins from the nine proteomic identification approaches for Gatto-TMT6 and Hultin-iTRAQ8, respectively. It can be observed that identification approaches based on a single database search tool generate varying numbers of identified proteins (386–449 proteins for Gatto-TMT6 and 2018–2305 proteins for Hultin-iTRAQ8), and in general are outnumbered by identification approaches based on multiple database search tools (452–460 proteins for Gatto-TMT6 and 2378–2555 proteins for Hultin-iTRAQ8). For both data sets, the last search result (Comet and X!Tandem searches in TPP followed by PeptideProphet, iProphet, and Mayu) generates the highest number of proteins, even higher than combined searches using Comet, X!Tandem, and MSGF+^46^ and validated by PeptideShaker; notably, Hultin-iTRAQ8 shows an increase of 7.4% or 177 proteins (from 2378 to 2555 proteins) being identified. The results suggest validation tools can be a significant factor affecting the coverage of the identified proteome. This phenomenon highlights an advantage of Multi-Q 2—the capability of accepting multiple database search results from several popular proteomic data analysis systems including TPP, PeptideShaker, and Proteome Discoverer. Because of the largest coverage, the identification results based on TPP were used by Multi-Q 2 to perform quantitation for further benchmark analysis.

### Comprehensive comparison of different algorithmic combinations used in Multi-Q 2 for quantitation

#### Overview of the comparison

To evaluate different algorithmic combinations of Multi-Q 2, we used the standard data sets Gatto-TMT6 and Hultin-iTRAQ8 since the ideal protein ratios are known and can be used as the ground truth. There are various algorithms in the workflow of Multi-Q 2, including four algorithms for peptide ratio calculation, six algorithms for protein ratio calculation, and two possibilities of RCC (enabled or disabled). In addition, we used seven different normalization approaches: no normalization, reporter ion level normalization only, peptide level normalization only, protein level normalization only, reporter ion plus peptide level normalization, reporter ion plus protein level normalization, and all three levels of normalization.

A large-scale systematic benchmark experiment was conducted on Gatto-TMT6 by exhaustively generating all 336 (4 × 6 × 7 × 2) possible combinations of different quantitation and normalization algorithms. Similarly, on Hultin-iTRAQ8 48 (4 × 6 × 2) algorithmic combinations were exhaustively generated with no normalization because the sample proportions across different channels in this data set are not of equal amount. Note that some of the combinations can have identical quantitation results at protein level regardless of any peptide ratio calculation method used. For example, if protein ratios are directly derived from PSM ratios, altering peptide ratio calculation algorithm does not change protein ratios.

#### Comparison of all possible algorithmic combinations in Multi-Q 2 on Gatto-TMT6

**Figure 2:**
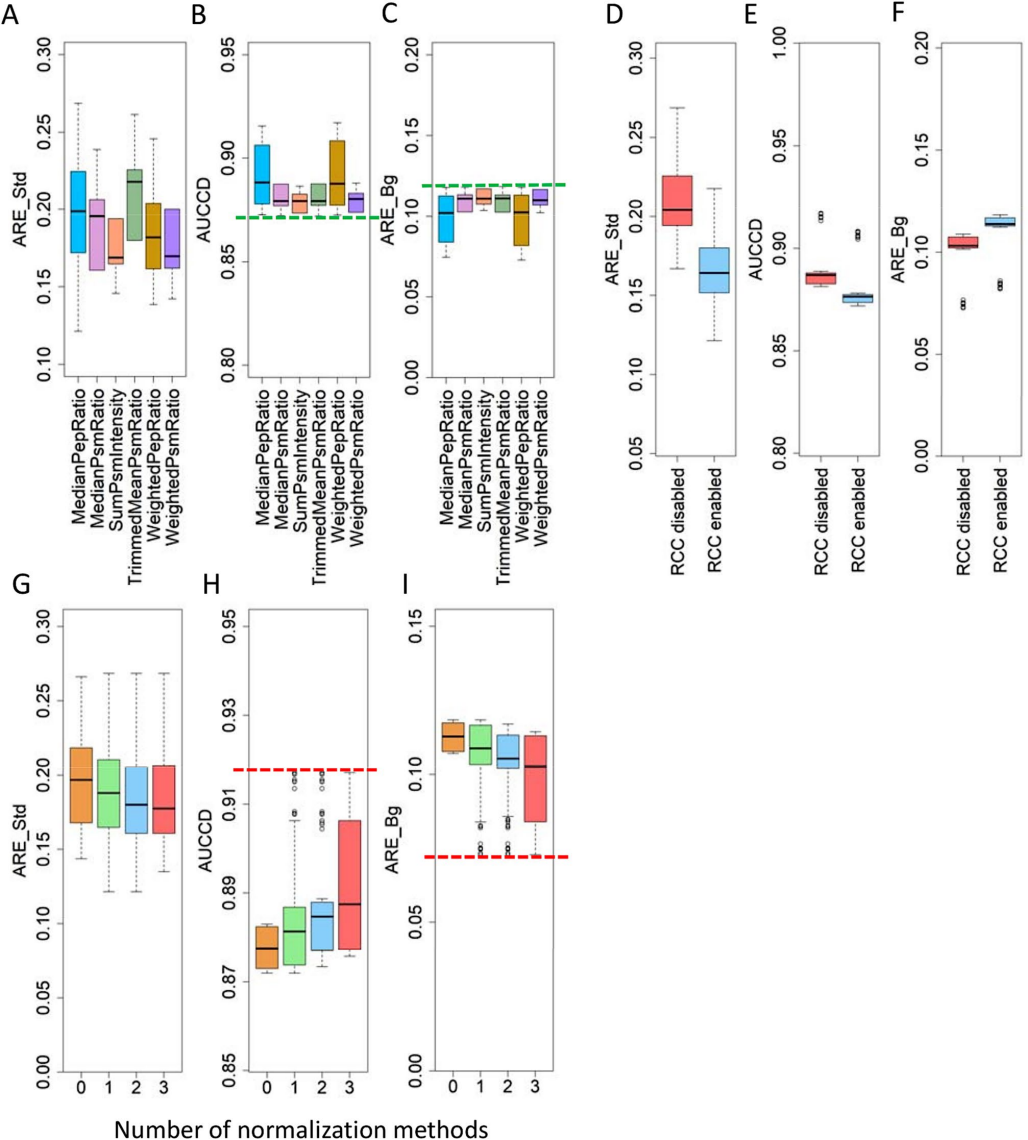
Summarizations of evaluation on algorithmic combinations for (**A**–**C**) different protein ratio calculation algorithms, (**D**–**F**) RCC enabled and disabled, and (**G**–**I**) the number of normalization methods based on Gatto-TMT6. Evaluation measures include average relative error of standard proteins (ARE_Std), area under the curve of coverage vs. deviation (AUCCD), and average relative error of background proteins (ARE_Bg). Each boxplot in panels A–C and D–F represents a summarization of 56 and 168 algorithmic combinations, respectively. Boxplots of 0, 1, 2, and 3 normalization methods in panels G-I correspond to summarizations of 48, 144, 96, and 48 algorithmic combinations, respectively.

Benchmark results of 336 algorithmic combinations on Gatto-TMT6 are shown in Supplementary Table S1. It can be observed that the top 20 algorithmic combinations evaluated by AUCCD and ARE have very close performances, suggesting that there are a group of algorithmic combinations rather than a single one capable of generating accurate quantitation results. We summarize the information in Supplementary Table S1 to compare the six protein ratio calculation algorithms, RCC enabled and disabled, and the number of normalization algorithms being used in Fig. 2. Figure 2A shows that algorithmic combinations using MedianPepRatio for protein ratio calculation have the lowest ARE_Std as evidenced by its lowest whisker. From Fig. 2B,C it is obvious the worst case of the six algorithms in terms of AUCCD and ARE_Bg are comparable, as indicated by the green dashed lines. Algorithmic combinations using MedianPepRatio and WeightedPepRatio have significantly larger interquartile range, leading to the best AUCCD and ARE_Bg. Interestingly, these are the only two methods that derive protein ratios from peptide ratios. The situation suggests an additional stage at peptide level can provide useful information which can increase the protein-level quantitation accuracy of background proteins. Enabling RCC produces considerable improvement (by 0.05, from 0.2 down to 0.15) in ARE_Std (Fig. 2D), but inevitably deteriorates AUCCD (Fig. 2E) and ARE_Bg (Fig. 2F) by 0.01–0.02 because RCC tends to pull protein ratios away from one. It can also be seen that algorithmic combinations with normalization achieve better AUCCD, ARE_Bg, and ARE_Std, compared to those without normalization. Using more normalization approaches (applied at different levels) in general improve AUCCD (Fig. 2H) and ARE_Bg (Fig. 2I) of different algorithmic combinations. However, the combinations with the lowest ARE_Bg and the highest AUCCD seem to be comparable for using one, two, and three normalization methods, as indicated by the red dashed lines in Fig. 2H,I. Applying any number of normalization methods creates a slight improvement in ARE_Std over the case without normalization, as shown in Fig. 2G.

When sorting Supplementary Table S1 by ARE_Std in ascending order, the method of using MedianPepRatio for protein ratio and LinearRegression for peptide ratio with RCC enabled (denoted as MedianPepRatio-LinearRegression-enRCC) yields the best performance, with an ARE_Std of 0.122 among 336 algorithmic combinations. Incorporating this method with different normalization strategies does not significantly affect the performance as long as normalization is enabled, as evidenced by the top five algorithmic combinations in the sorted table (with ARE ranging from 0.122 to 0.135). When sorting Supplementary Table S1 by AUCCD in descending order or by ARE_Bg in ascending order, the method of using WeightedPepRatio for protein ratio and WeightedPsmRatio for peptide ratio with RCC disabled (denoted as WeightedPepRatio-WeightedPsmRatio-disRCC) is one of the best algorithmic combinations, generating an AUCCD of 0.917 and an ARE_Bg of 0.073. Again, these measures are insensitive to different normalization strategies as long as normalization is enabled, as evidenced by the top three algorithmic combinations in the sorted table. The results reveal MedianPepRatio-LinearRegression-enRCC better fits protein ratios of significant fold changes (the four standard proteins’ ratios ranging from 0.1 to 10) while it inevitably sacrifices the accuracy for protein ratios with an ideal value of one. WeightedPepRatio-WeightedPsmRatio-disRCC tends to compress protein ratios towards one and better fits the ratios of background proteins (yielding higher AUCCD and lower ARE_Bg) while sacrificing the accuracy for proteins with significant fold changes (yielding higher ARE_Std).

#### Comparison of all possible algorithmic combinations in Multi-Q 2 on Hultin-iTRAQ8

Benchmark results of the 48 algorithmic combinations on Hultin-iTRAQ8 are reported in Supplementary Table S2. It can be observed the best algorithmic combination for background proteins of Gatto-TMT6, WeightedPepRatio-WeightedPsmRatio-disRCC, yields an AUCCD of 0.945 and an ARE of 0.091 on Hultin-iTRAQ8 and is among one of the best algorithmic combinations evaluated by both measures. The algorithmic combination of MedianPepRatio-LinearRegression-enRCC yields an AUCCD of 0.934 (rank 33/48) and an ARE of 0.113 (rank 32/48). The relatively worse performance can be anticipated because MedianPepRatio-LinearRegression-enRCC performs better for protein ratios with significant fold changes, which are not present in this data set. To further summarize the information in Supplementary Table S2, boxplots are generated and reported in Supplementary Figs. S3 and S4. Supplementary Fig. S3 indicates that all the six algorithms for protein ratio calculation have similar boxplot ranges for both AUCCD and ARE. That being said, the highest AUCCD and the lowest ARE are achieved by methods based on MedianPepRatio, WeightedPepRatio, and WeightedPsmRatio, as illustrated by the red dashed lines in Supplementary Fig. S3A and B. Supplementary Fig. S4 shows that enabling RCC results in slightly lower AUCCD and higher ARE, which is in good agreement with the observations from Gatto-TMT6, as shown in Fig. 2E,F. As the ideal protein ratios in this data set are either 1 or 2 (smaller fold changes), we can therefore conclude that RCC slightly deteriorates ARE for proteins of smaller fold changes, despite its improved accuracy for proteins of larger fold changes.

#### Comparison of 12 algorithmic combinations on NCI-7

**Figure 3:**
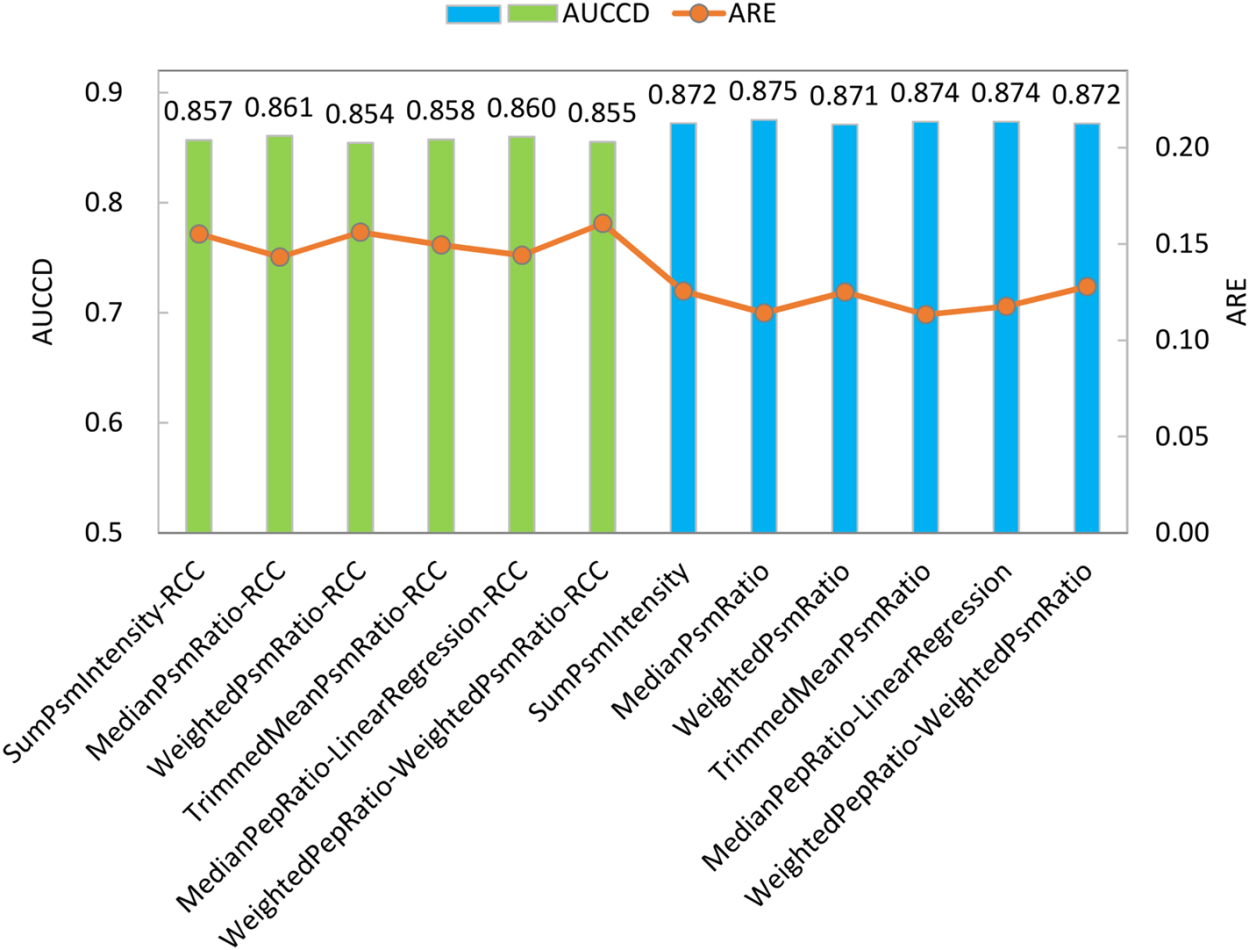
Area under the curve of coverage vs deviation (AUCCD, primary y-axis) and average relative error (ARE, secondary y-axis) of the 12 algorithmic combinations for NCI-7. The green and blue bars correspond to methods with RCC enabled and disabled, respectively.

We used Multi-Q 2 to quantify a total of 10,524 proteins (with no missing value in any reporter ion) in NCI-7. Six protein ratio calculation methods were used for benchmark analysis, where the two methods based on peptide ratios are particularly chosen to be MedianPepRatio-LinearRegression and WeightedPepRatio-WeightedPsmRatio based on our previous analyses on Gatto-TMT6 and Hultin-iTRAQ8. For all the six methods, RCC can be enabled or disabled, resulting in 12 different algorithmic combinations. Normalization was disabled because of the unequal abundance ratios (i.e., 1:1:0.5:1:1:0.5:1:1:0.5:1) among channels. Performance evaluation of the quantitation results from the 12 algorithmic combinations is illustrated in Fig. 3. It can be seen different protein ratio calculation algorithms produce slight differences in terms of AUCCD and ARE, whereas enabling RCC (green bars in Fig. 3) leads to a decrease of AUCCD ranging from 0.014 to 0.017 and an increase of ARE ranging from 0.026 to 0.037 (for the same protein ratio calculation algorithm). This phenomenon is consistent to the observations from Hultin-iTRAQ8 and from background proteins of Gatto-TMT6: enabling RCC alleviates ratio compression problem at the cost of lowering the quantitation accuracy for background proteins with fold changes close to 1. Nevertheless, switching protein ratio calculation algorithms has relatively smaller impact to overall performance for NCI-7 possibly because (1) wet-lab experimental procedures were rigorously performed, (2) the acquired MS spectra have good signal-to-noise ratio and are of high quality, and (3) the data set does not have proteins of large fold changes for which different algorithms can sometimes produce quite different estimated protein ratios.

#### Summary of performance evaluation based on different algorithmic combinations

The experiment results show that several algorithmic combinations, instead of a single combination, can achieve the best performance. In general, the algorithmic combination most suitable for quantifying proteins of significant fold changes (i.e., differentially expressed proteins) sacrifices its accuracy for proteins of insignificant fold changes (i.e., background proteins). Enabling RCC does to some extent remedy ratio compression, but inevitably decreases the quantitation accuracy for background proteins because it tends to pull protein ratios away from one. To quantify a data set with equal amounts of samples across different channels, algorithmic combinations with normalization achieve better AUCCD, ARE_Bg, and ARE_Std compared to those without normalization, as shown in the results of Gatto-TMT6. Using one, two, and three different normalization methods can all achieve the lowest ARE_Bg and the highest AUCCD. For NCI-7, altering protein ratio calculation algorithm has marginal impact on AUCCD and ARE. Enabling RCC does lead to a slight decrease in AUCCD and a slight increase in ARE, both are in good agreement with the observations of background proteins from Gatto-TMT6 and Hultin-iTRAQ8.

### Performance comparison of Multi-Q 2 and existing tools using Gatto-TMT6

To evaluate the performance of Multi-Q 2, we compared its performance with those of PatternLab, MaxQuant, and Libra on Gatto-TMT6. In addition to AUCCD and ARE, we use root mean square error (RMSE), mean, and standard deviation (SD) to evaluate protein ratios of background proteins.

For quantitation, Libra uses the same identification results as Multi-Q 2 (i.e., Comet and X!Tandem searches on TPP followed by PeptideProphet, iProphet and Mayu), and PatternLab and MaxQuant use their built-in Comet and Andromeda, respectively, for database searches, followed by their own statistical validation algorithms. The numbers of quantifiable proteins of Multi-Q 2, PatternLab, MaxQuant, and Libra are 456, 406, 418, and 440, respectively. Multi-Q 2 has the largest coverage because it benefits from the use of multiple database search engines and its flexibility in supporting various protein and peptide validation systems, as mentioned earlier.

**Figure 4:**
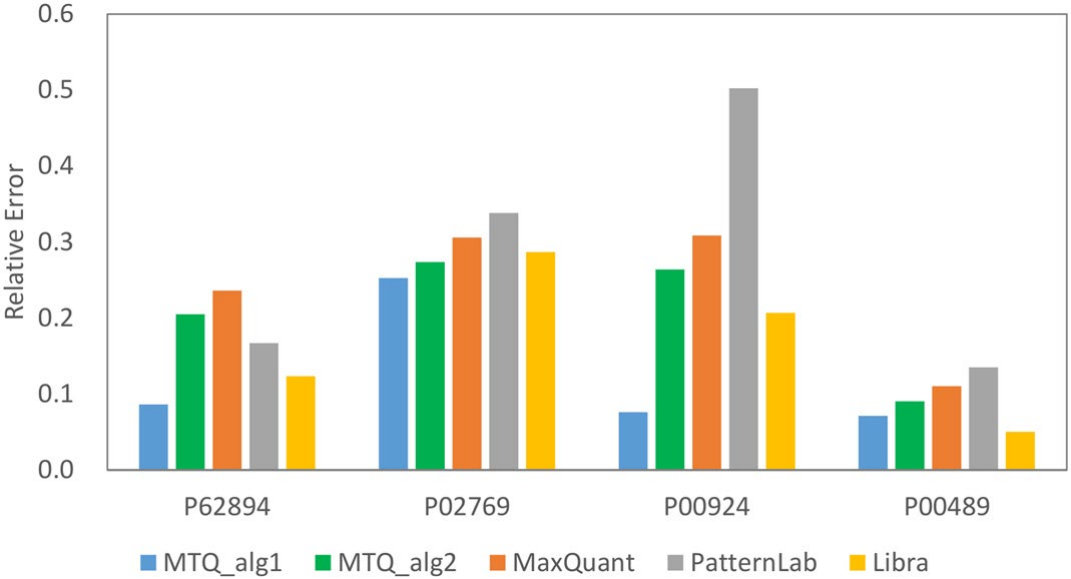
Comparison of relative errors of the four standard proteins of Gatto-TMT6 among different software tools.

No single algorithmic combination is the best for all situations under all evaluation measures according to the previous analyses. For the purpose of performance comparison, two algorithmic combinations of Multi-Q 2 were selected. The first, denoted by MTQ_alg1, stands for MedianPepRatio-LinearRegression with normalization at reporter ion level and RCC enabled (one of the best algorithmic combinations for standard proteins in Gatto-TMT6). The second, denoted by MTQ_alg2, stands for WeightedPepRatio-WeightedPsmRatio with normalization at peptide level and RCC disabled (one of the best algorithmic combinations for background proteins in Gatto-TMT6). Relative errors of the four standard proteins for the compared tools are illustrated in Fig. 4, and ARE_Std for MTQ_alg1, MTQ_alg2, MaxQuant, PatternLab, and Libra are 0.122, 0.208, 0.240, 0.286, and 0.167, respectively. It is evident that our systematic evaluation of quantitation algorithms for quantifying standard proteins leads to significant improvement of MTQ_alg1 over the compared tools. Interestingly, MTQ_alg2, the one more suitable for quantifying background proteins according to our analysis, compares favorably over MaxQuant and PatternLab in terms of ARE_Std as well. It is worth noting that the improvement of ARE_Std in Multi-Q 2 comes partly from the use of RCC, since disabling it leads to an overall increase of 0.056 in ARE_Std, as shown in Supplementary Fig. S5. We further evaluated the four tools on the 372 background proteins commonly quantifiable by all of them, and the results are summarized in Table 1. MTQ_alg2 yields the highest AUCCD and the lowest ARE_Bg and RMSE among all the compared software tools; its mean of protein ratios is closest to the theoretical value of 1, and its standard deviation (SD) is the smallest. It is obvious that MTQ_alg1 does not perform as well as MTQ_alg2 in this evaluation. The results on MTQ_alg1 and MTQ_alg2 again highlight an important fact: the quantitation algorithms that better fit significant fold changes could have lower accuracy for fitting insignificant fold changes (i.e., without differential expression), and vice versa. It is thus beneficial for a software tool to provide the flexibility of customizing the quantitation algorithms for different purposes.

**Table 1:** Performance comparison of the four software tools on the 372 commonly quantifiable background proteins of Gatto-TMT6.

	MTQ_alg1	MTQ_alg2	MaxQuant	PatternLab	Libra
AUCCD	0.886	0.928*	0.895	0.909	0.902
ARE_Bg	0.105	**0.062**	0.095	0.081	0.088
RMSE	0.139	**0.105**	0.145	0.131	0.125
Mean	0.934	**1.006**	1.055	1.037	0.972
SD	0.122	**0.105**	0.135	0.126	0.122

*Boldface indicates the best measure among the four tools.

### Performance comparison of Multi-Q 2 and existing tools using Hultin-iTRAQ8

Multi-Q 2, PatternLab, MaxQuant, and Libra quantified 2528, 2285, 2315, and 2486 proteins, respectively. Again, Multi-Q 2 has significantly larger coverage in quantifiable proteins than MaxQuant and PatternLab because it benefits from the use of multiple search engines (Comet and X!Tandem searches in TPP followed by PeptideProphet, iProphet, and Mayu). We then evaluated the four tools on the 2023 commonly quantifiable proteins, and the results are summarized in Table 2. MTQ_alg2 yields an AUCCD of 0.905, an ARE of 0.086, and a RMSE of 0.218, all of which are the best among all the tools; MTQ_alg1 achieves the second best in terms of these three measures. MTQ_alg1 yields the smallest standard deviation for protein ratios with ideal values of 2 (SD2). Libra generates protein ratios with their mean closest to the theoretical value of either 1 (Mean1) or 2 (Mean2). PatternLab generates the smallest standard deviation for protein ratios with ideal values of 1 (SD1). We further performed pairwise comparisons of protein fold changes calculated by MTQ_alg2 and the other three tools (MaxQuant, PatternLab, and Libra); the results are shown in Supplementary Fig. S6. All of them show very high correlation (R^2^ between 0.93 and 0.96; slope between 0.88 and 0.96) though a small portion of protein ratios are distant to the diagonal.

**Table 2:** Evaluation measures for 2023 commonly quantifiable proteins of the four software tools on Hultin-iTRAQ8.

	MTQ_alg1	MTQ_alg2	MaxQuant	Libra	PatternLab
AUCCD	0.901	0.905*	0.889	0.894	0.899
ARE	0.090	**0.086**	0.102	0.094	0.091
RMSE	0.220	**0.218**	0.243	0.228	0.226
Mean1^#^	0.955	0.964	1.102	**0.989**	0.966
Mean2	1.964	1.981	2.099	**1.993**	1.970
SD1	0.155	0.148	0.148	0.156	**0.136**
SD2	**0.253**	0.256	0.263	0.269	0.271

*Boldface indicates the best measure among the tools.

^#^Since this standard data set has an ideal protein ratio of either 1 or 2, we use Mean1 and SD1 to represent mean and standard deviation of protein ratios with an ideal value of 1, and use Mean2 and SD2 to represent mean and standard deviation of protein ratios with an ideal value of 2.

### Remarks on algorithmic combinations

It is evident no single algorithmic combination can be claimed to be the best for all the situations. Even though the two algorithmic combinations, MTQ_alg1 and MTQ_alg2, were selected from rigorous experiments using various evaluation measures, there is no guarantee the two are the best for other data sets. The best algorithmic combination of a given data set can be dependent on a number of factors including instrument, spectrum quality, the complexity of samples, the definition of ratios (numerators and denominators), and evaluation measures. Optimization of algorithmic combinations or providing guidelines for the selection of quantitation algorithms for a given data set is a complicated issue and is beyond the scope of this study. Nevertheless, compared to other tools mostly based on a fixed quantitation method, the flexibility of Multi-Q 2 provides clear advantage in quantitation accuracy. The previous benchmark analyses demonstrate that Multi-Q 2 can outperform existing tools with proper selection of algorithmic combinations.

### Further evaluation of Multi-Q 2 on differentially expressed proteins of Chen-iTRAQ8

When proteins are differentially expressed in different states, e.g., tumor vs. normal tissue samples, acquiring a list of candidate biomarkers, namely, proteins of high fold changes across different states, is usually crucial for further biological discovery. Thus in this subsection, we further use Chen-iTRAQ8 to evaluate different algorithmic combinations of Multi-Q 2 in search of differentially expressed (temperature-dependent) proteins, which are defined as proteins with at least a ratio above 1.5 (see Supplementary Text S1 for more details).

A total of 1,064 proteins are quantifiable (without missing value in any reporter ion) by Multi-Q 2. Twelve algorithmic combinations of six protein ratio calculation methods used for NCI-7 with RCC enabled and disabled are used to analyze Chen-iTRAQ8 for finding temperature-dependent proteins. Normalization is applied at reporter ion level. The number of temperature-dependent proteins of these methods are shown in Supplementary Fig. S7. It can be seen that enabling RCC increases the number of temperature-dependent proteins regardless of the quantitation algorithms, since more protein ratios are pulled away from 1 and more proteins satisfy the criterion of temperature-dependent protein. SumPsmIntensity and WeightedPsmRatio outnumber others significantly, suggesting that they may be more sensitive but, on the other hand, are likely to produce more false positives as well.

**Figure 5:**
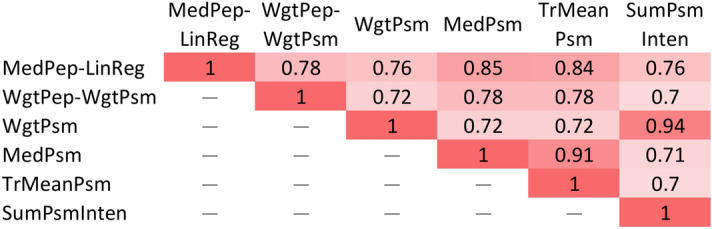
Pairwise similarities of (potential) temperature-dependent proteins between the 6 algorithmic combinations with RCC. Deep color indicates higher similarity. MedPep-LinReg stands for MedianPepRatio-LinearRegression; WgtPep-WgtPsm stands for WeightedPepRatio-WeightedPsmRatio; WgtPsm stands for WeightedPsmRatio; MedPsm stands for MedianPsmRatio; TrMeanPsm stands for TrimmedMeanPsmRatio; SumPsmInten stands for SumPsmIntensity.

We then evaluated the similarity of two lists of temperature-dependent proteins obtained from any pair of the six algorithmic combinations with RCC enabled by Jaccard index^51^, defined as the size of the intersection divided by the size of the union of the two lists. The results are illustrated in Fig. 5. It is observed that WeightedPsmRatio shares a high similarity of 0.94 with SumPsmIntensity, but a much lower similarity of 0.72 with MedianPsmRatio. In contrast, MedianPsmRatio shares a high similarity of 0.91 with TrimmedMeanPsmRatio. The results are logical because both WeightedPsmRatio and SumPsmIntensity tend to assign higher weights for PSMs of larger reporter ion intensities for calculating protein ratios. In contrast, both MedianPsmRatio and TrimmedMeanPsmRatio tend to eliminate outliers of PSM ratios without considering the weight of a PSM. Since different algorithmic combinations can yield quite different lists of candidate biomarkers and from our previous analyses there is still no single algorithmic combination that outperforms others in all evaluation measures under all the circumstances, it is suggested that users apply simultaneously algorithmic combinations of complementary rationales, for example, WeightedPsmRatio and MedianPsmRatio, while searching for biomarkers. The union of candidate biomarker lists from complementary algorithmic combinations can be considered to improve sensitivity. On the other hand, considering the common proteins from candidate biomarker lists of complementary algorithmic combinations and ignoring the rest can possibly improve precision.

Furthermore, examining the pairwise similarity of algorithmic combinations with RCC disabled, we observed overall decreases (ranging from 0.01 to 0.1) in all the pairwise similarities except for SumPsmIntensity vs WeightedPsmRatio, as illustrated in Supplementary Fig. S8. For example, the pairwise similarity between TrimmedMeanPsmRatio and WeightedPsmRatio decreases from 0.72 to 0.62 when disabling RCC. Supplementary Fig. S9 shows the Venn diagrams of these two algorithms with RCC enabled (Supplementary Fig. S9A) and disabled (Supplementary Fig. S9B). It can be observed that the number of overlapping temperature-dependent proteins increases from 378 to 469 (around 24% increase) when RCC is enabled. The phenomenon indicates that enabling RCC in general yields more consistency, namely, higher Jaccard index, among different quantitation algorithms. In addition, as shown above, RCC can increase the number of proteins of fold changes above 1.5. These observations suggest that RCC can be useful in searching for proteins of significant fold changes.

### Graphical user interfaces of Multi-Q 2

Multi-Q 2 was implemented with Visual Studio C# and can be run on the Windows platforms (Windows 7, 8, and 10; Windows Server 2008, 2012, and 2016) with a prerequisite of .net framework 4.0 or newer versions. A quantitation wizard, as shown in Supplementary Fig. S10, provides step-by-step procedures for users to set up parameters and input files for quantitation. Quantitation results are displayed in the main window of Multi-Q 2 as illustrated in Supplementary Fig. S11.

In addition to the main window, Multi-Q 2 supports a heatmap module, through which users can cluster proteins of similar ratios using different clustering algorithms—hierarchical clustering, K-means, and fuzzy C-means—based on different distance measures—Euclidean distance, Manhattan distance, Pearson’s correlation coefficient, and covariance. An example of heatmap is illustrated in Supplementary Fig. S12. Multi-Q 2 can summarize distributions of reporter ion intensities from the identified PSMs as boxplots illustrated in Supplementary Fig. S13A, or as bar charts illustrated in Supplementary Fig. S13B. Similarly, it can summarize distributions of protein ratios as a boxplot illustrated in Supplementary Fig. S14A, or as a bar chart illustrated in Supplementary Fig. S14B.

## Conclusion

We present Multi-Q 2, a versatile software tool for isobaric labeling quantitation. Multi-Q 2 supports identification results from TPP, PeptideShaker, and Proteome Discoverer, offering up to 12% improvement in coverage compared with MaxQuant and PatternLab. Multi-Q 2 is implemented with popular quantitation, normalization, and ratio compression correction algorithms, supporting up to 336 algorithmic combinations. Our benchmark analyses show that Multi-Q 2 is capable of generating the largest quantitation coverage and improved quantitation accuracy compared with state-of-the-art software solutions. More importantly, we show that there is still no single algorithmic combination that outperforms others in all evaluation measures under all circumstances. It is therefore beneficial for a software tool to provide the flexibility of customizing quantitation algorithms. We further demonstrate that using algorithmic combinations of complementary rationale simultaneously in search of candidate biomarkers can be a reasonable strategy to improve either sensitivity or precision. Multi-Q 2 is a standalone and installation-free Windows program. It provides friendly graphical interfaces for users to perform quantitation and follow-up analysis, such as clustering and heatmap visualization. We believe that Multi-Q 2 can be of great help in searching for potential biomarkers from complex proteomic samples.

## Supplementary information

Supplementary Table S1.

Supplementary Table S2.

Supplementary text and figures.

## Data Availability

Multi-Q 2 executable files, sample data sets, and user manual are freely available at http://ms.iis.sinica.edu.tw/COmics/Software_Multi-Q2.html.
