# Supplementary material for: Multi-Q 2 software facilitates isobaric labeling quantitation analysis with improved accuracy and coverage
Source: Sci Rep. 2021 Jan 26;11:2233. doi: 10.1038/s41598-021-81740-4 (PMC7838301; doi:10.1038/s41598-021-81740-4)
Supplement: Supplementary file 3 — Supplementary text and figures. [file 41598_2021_81740_MOESM3_ESM.pdf]

Multi-Q 2 software facilitates isobaric labeling quantitation analysis with improved accuracy and coverage

Ching-Tai Chen,[1*] Jen-Hung Wang,[123] Cheng-Wei Cheng,[4] Wei-Che Hsu,[1] Chu-Ling Ko,[5] Wai-Kok Choong,[1] Ting-Yi Sung[1*]

[1]Institute of Information Science, Academia Sinica, Taipei 115, Taiwan

[2]Bioinformatics Program, Taiwan International Graduate Program, Academia Sinica, Taipei 115, Taiwan

[3]Institute of Biomedical Informatics, National Yang-Ming University, Taipei 112, Taiwan

[4]Genomics Research Center, Academia Sinica, Taipei 115, Taiwan

[5]Department of Computer Science and Engineering, University of California San Diego, La Jolla, California 92093, United States

[*]Correspondence:
Prof. Ting-Yi Sung
Institute of Information Science
128 Academia Road, Section 2, Nankang, Taipei 115, Taiwan
Tel: +886-2-2788-3799 ext. 1711
Fax: +886-2-2651-8660
Email: tsung@iis.sinica.edu.tw

Dr. Ching-Tai Chen
Institute of Information Science
128 Academia Road, Section 2, Nankang, Taipei 115, Taiwan
Tel: +886-2-2788-3799 ext. 2352
Fax: +886-2-2651-8660
Email: ctchen@iis.sinica.edu.tw

Table of Content

Text S1. Data sets 3
Figure S1. Numbers of identified proteins with different database search methods for Gatto-TMT6 data set. 5
Figure S2. Numbers of identified proteins with different database search methods for Hultin-iTRAQ8 data set. 6
Figure S3. Summarizations of evaluation on algorithmic combinations for different protein ratio calculation algorithms. 7
Figure S4. Summarizations of evaluation on algorithmic combinations with ratio compression correction (RCC) enabled and disabled 8
Figure S6. Correlations of calculated protein fold changes on Hutlin-iTRAQ8. 10
Figure S7. The number of temperature-dependent proteins in Chen-iTRAQ8 obtained by Multi-Q 2 using different algorithmic combinations. 11
Figure S8. Pairwise similarity of temperature-dependent proteins in Chen-iTRAQ8 using the six algorithmic combinations without RCC. 12
Figure S9. Venn diagram of temperature-dependent proteins in Chen-iTRAQ8 obtained by Multi-Q 2 using WeightedPsmRatio and TrimmedMeanPsmRatio. 13
Figure S10. Screenshots of Multi-Q 2 quantitation wizard. 15
Figure S11. Main window of Multi-Q 2. 16
Figure S12. Heatmap module in Multi-Q 2. 18
Figure S13. Screenshots of Multi-Q 2 regarding distributions of reporter ion intensities. 19
Figure S14. Screenshots of Multi-Q 2 regarding distributions of protein ratios. 20

Text S1. Data sets

The first data set, denoted as Gatto-TMT6, consists of TMT-6 samples of *Erwinia carotovora* lysate and was downloaded from the ProteomeXchange Consortium[1] with the identifier PXD000001.[2] The data set has background proportions of samples at 1:1:1:1:1:1 and four spike-in proteins with their proportions at 10:5:2.5:1:2.5:10 (yeast enolase, ENO), 1:2.5:5:10:5:1 (bovin serum albumin, BSA), 2:2:2:2:1:1 (rabbit glycogen phosphorylase, PHO) and 1:1:1:1:1:2 (bovin cytochrome c, CYT). Five protein ratios (r1 to r5) are defined by taking the first channel as the denominator and the rest as the numerators.

The second data set, denoted as Hultin-iTRAQ8, consists of iTRAQ-8 samples of human A549 cell lysate and was downloaded from the ProteomeXchange Consortium with the identifier PXD000418.[3] Different amounts of cell lysates are mixed with proportions at 2:2:1:1:2:2:1:1. Seven protein ratios (r1 to r7) are defined by taking the last channel as the denominator and the rest as the numerators. Both data sets were acquired from an LTQ OrbitrapVelos mass spectrometer (Thermo Fisher Scientific).

The third data set, named NCI-7, was downloaded from the CPTAC Data Portal.[4] It consists of TMT-10 samples from seven cancer cell lines including NCI-H23, RPMI-8226, T47D, A549, COLO205, NCI-H226, and CCRF-CEM. The samples are run on an Orbitrap Lumos Fusion system (Thermo Fisher Scientific). Channels one to three were mixed with portions at 1:1:0.5 from the first replicate of the digested mixture. Similar approaches were performed for channels four to six and seven to nine from the second and the third replicates, respectively. The last channel corresponds to a pooled reference generated with equal amounts of protein from each individual cell line. By taking the last channel as the denominator and the other nine as the numerators, we have nine ratios with theoretical values of either 1 or 0.5.

The fourth data set, named Chen-iTRAQ8, consists of iTRAQ-8 samples of *anaerobic thermophilic eubacterium* (*T. tengcongensis*) and was obtained from the ProteomeXchange Consortium with the identifier PXD000264.[5] The data set was generated with LTQ Orbitrap Velos mass spectrometer (Thermo Fisher Scientific). Four channels, 114, 115, 116, and 117 were used in the experiment, representing samples at temperatures of 55, 65, 75, and 80 °C, respectively. In this study, protein abundance at 55 °C is used as the reference. A protein at any of the other three temperatures showing a large abundance change against the reference is regarded as a temperature-dependent protein. Accordingly, channel 114 is used as the denominator and the rest as the numerators; fold change cutoff is set to 1.5 as suggested in the original study.

References:

(1) Deutsch, E. W.; Csordas, A.; Sun, Z.; Jarnuczak, A.; Perez-Riverol, Y.; Ternent, T.; Campbell, D. S.; Bernal-Llinares, M.; Okuda, S.; Kawano, S.; Moritz, R. L.; Carver, J. J.; Wang, M.; Ishihama, Y.; Bandeira, N.; Hermjakob, H.; Vizcaíno, J. A. The ProteomeXchange Consortium in 2017: Supporting the Cultural Change in Proteomics Public Data Deposition. *Nucleic Acids Res* 2017, 45 (D1), D1100–D1106. https://doi.org/10.1093/nar/gkw936.

(2) Gatto, L.; Christoforou, A. Using R and Bioconductor for Proteomics Data Analysis. *Biochimica et Biophysica Acta (BBA) - Proteins and Proteomics* 2014, 1844 (1, Part A), 42–51. https://doi.org/10.1016/j.bbapap.2013.04.032.

(3) Hultin-Rosenberg, L.; Forshed, J.; Branca, R. M. M.; Lehtiö, J.; Johansson, H. J. Defining, Comparing, and Improv-ing ITRAQ Quantification in Mass Spectrometry Proteomics Data. *Mol Cell Proteomics* 2013, 12 (7), 2021–2031. https://doi.org/10.1074/mcp.M112.021592.

(4) Edwards, N. J.; Oberti, M.; Thangudu, R. R.; Cai, S.; McGarvey, P. B.; Jacob, S.; Madhavan, S.; Ketchum, K. A. The CPTAC Data Portal: A Resource for Cancer Proteomics Re-search. *J. Proteome Res.* 2015, 14 (6), 2707–2713. https://doi.org/10.1021/pr501254j.

(5) Chen, Z.; Wen, B.; Wang, Q.; Tong, W.; Guo, J.; Bai, X.; Zhao, J.; Sun, Y.; Tang, Q.; Lin, Z.; Lin, L.; Liu, S. Quantitative Proteomics Reveals the Temperature-Dependent Proteins Encoded by a Series of Cluster Genes in Thermoanaerobacter Tengcongensis. *Mol Cell Proteomics* 2013, 12 (8), 2266–2277. https://doi.org/10.1074/mcp.M112.025817.

Figure S1.

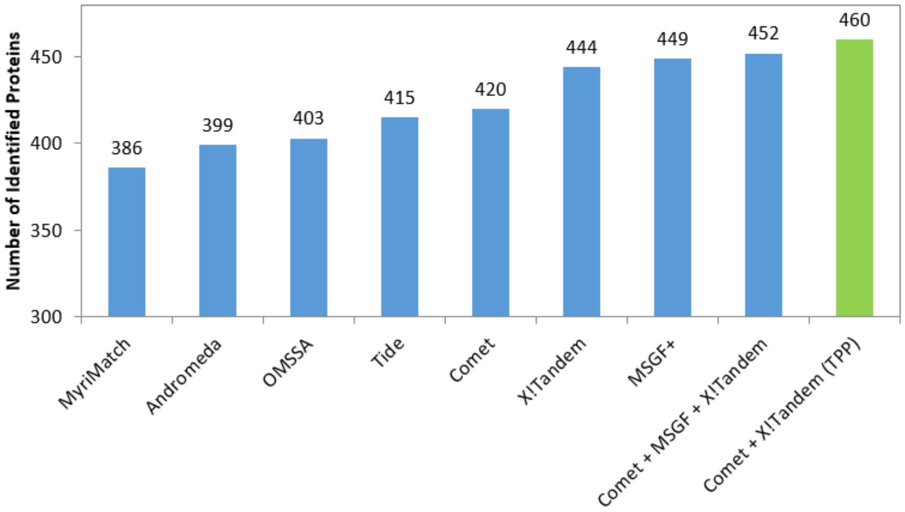

Figure S1. Numbers of identified proteins with different database search methods for Gatto-TMT6 data set.

Blue bars indicate methods based on the search engines from SearchGUI and validated with PeptideShaker. The green bar indicates Comet and X!Tandem searches from Trans-Proteomic Pipeline and validated with PeptideProphet, iProphet, and Mayu. All of the identification results follow a 1% false discovery rate at peptide and protein levels.

Figure S2.

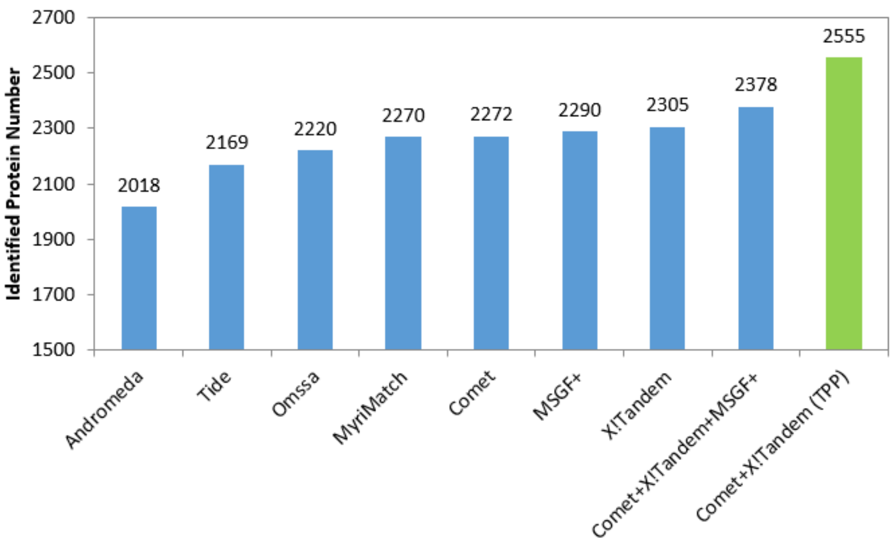

Figure S2. Numbers of identified proteins with different database search methods for Hultin-iTRAQ8 data set.

Blue bars indicate methods based on the search engines from SearchGUI and validated with PeptideShaker. The green bar indicates Comet and X!Tandem searches from Trans-Proteomic Pipeline and validated with PeptideProphet, iProphet, and Mayu. All of the identification results follow a 1% false discovery rate at peptide and protein levels.

Figure S3.

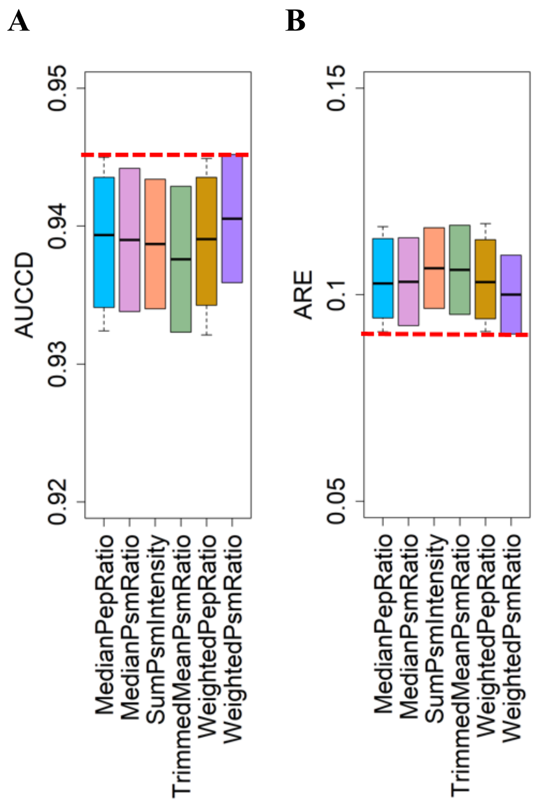

Figure S3. Summarizations of evaluation on algorithmic combinations for different protein ratio calculation algorithms.

Each boxplot represents a summarization of 8 algorithmic combinations evaluated with (A) area under the curve of coverage vs. deviation (AUCCD), and (B) average relative error (ARE) of all proteins from Hultin-iTRAQ8.

Figure S4.

A **B**

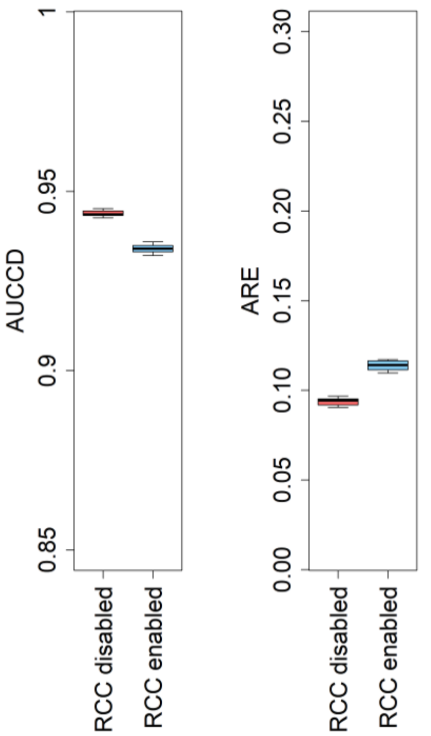

Figure S4. Summarizations of evaluation on algorithmic combinations with ratio compression correction (RCC) enabled and disabled.

Each boxplot represents a summarization of 168 algorithmic combinations evaluated with (A) area under the curve of coverage vs. deviation (AUCCD), and (B) average relative error (ARE) of all proteins from Hultin-iTRAQ8.

Figure S5.

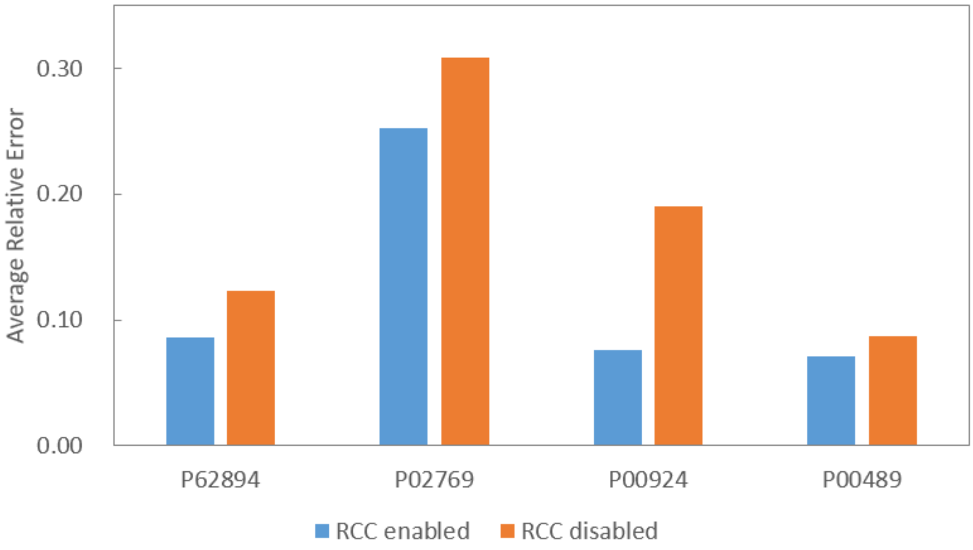

Figure S5. Comparison of the average relative error of the four standard proteins with RCC (Ratio Compression Correction) enabled or disabled.

Details of the quantitation algorithm are: MedianPepRatio for protein ratio, LinearRegression for peptide ratio, normalization at reporter ion level, with RCC enabled or disabled. The four standard proteins are from Gatto-TMT6.

Figure S6.

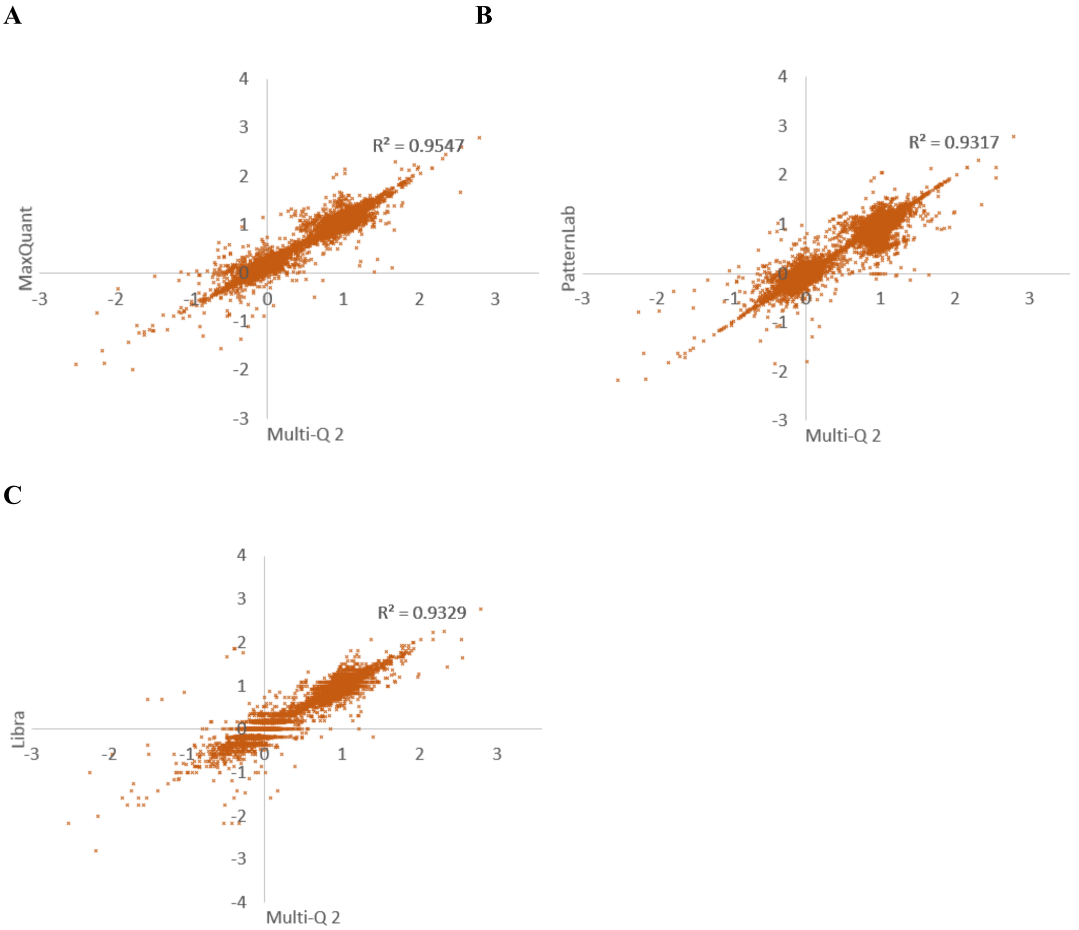

Figure S6. Correlations of calculated protein fold changes on Hutlin-iTRAQ8.

(A) Multi-Q 2 v.s. MaxQuant, (B) Multi-Q 2 v.s. PatternLab, (C) Multi-Q 2 v.s. Libra. Each point in the figure represents a single protein ratio. The slopes of panel A, B, and C are 0.88, 0.96, and 0.95, respectively.

Figure S7.

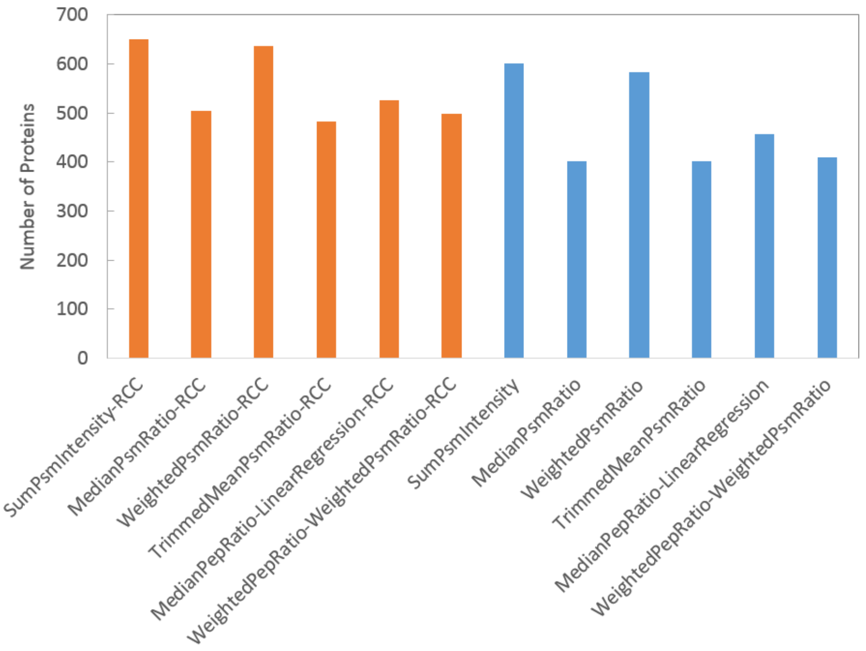

Figure S7. The number of temperature-dependent proteins in Chen-iTRAQ8 obtained by Multi-Q 2 using different algorithmic combinations.

Figure S8.

	MedPep-LinReg	WgtPep-WgtPsm	WgtPsm	MedPsm	TrMeanPsm	SumPsmInten
MedPep-LinReg	1	0.77	0.69	0.78	0.78	0.69
WgtPep-WgtPsm	—	1	0.64	0.74	0.74	0.62
WgtPsm	—	—	1	0.61	0.62	0.96
MedPsm	—	—	—	1	0.88	0.61
TrMeanPsm	—	—	—	—	1	0.62
SumPsmInten	—	—	—	—	—	1

Figure S8. Pairwise similarity of temperature-dependent proteins in Chen-iTRAQ8 using the six algorithmic combinations without RCC.

Deeper color indicates higher similarity. MedPep-LinReg stands for MedianPeptideRatio-LinearRegression, WgtPep-WgtPsm for WeightedPepRatio-WeightedPsmRatio, WgtPsm for WeightedPsmRatio, MedPsm for MedianPsmRatio, TrMeanPsm for TrimmedMeanPsmRatio, and SumPsmInten for SumPsmIntensity. The similarity is calculated with Jaccard index.

Figure S9.

A

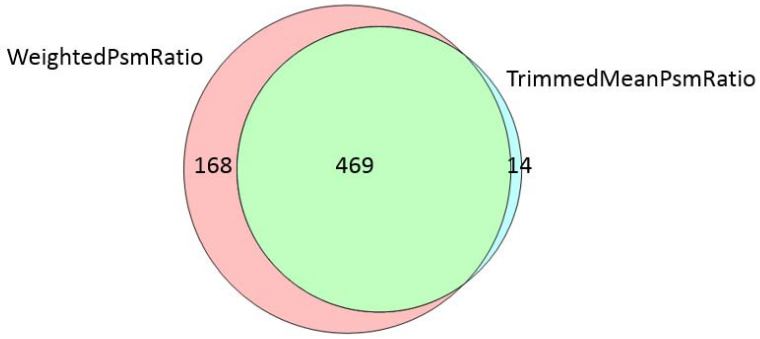

B

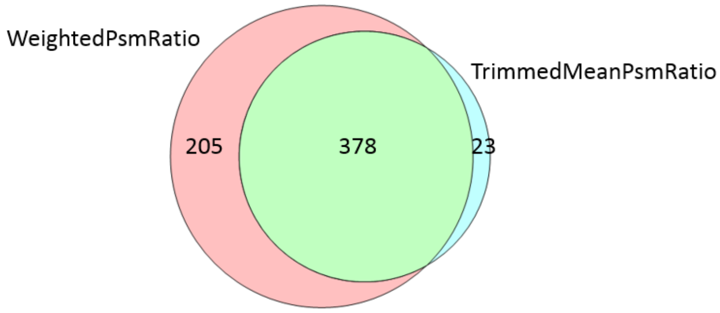

Figure S9. Venn diagram of temperature-dependent proteins in Chen-iTRAQ8 obtained by Multi-Q 2 using WeightedPsmRatio and TrimmedMeanPsmRatio.

(A) RCC enabled for both algorithmic combinations (pairwise similarity of 0.72); (B) RCC disabled for both algorithmic combinations (pairwise similarity of 0.62).

Figure S10.

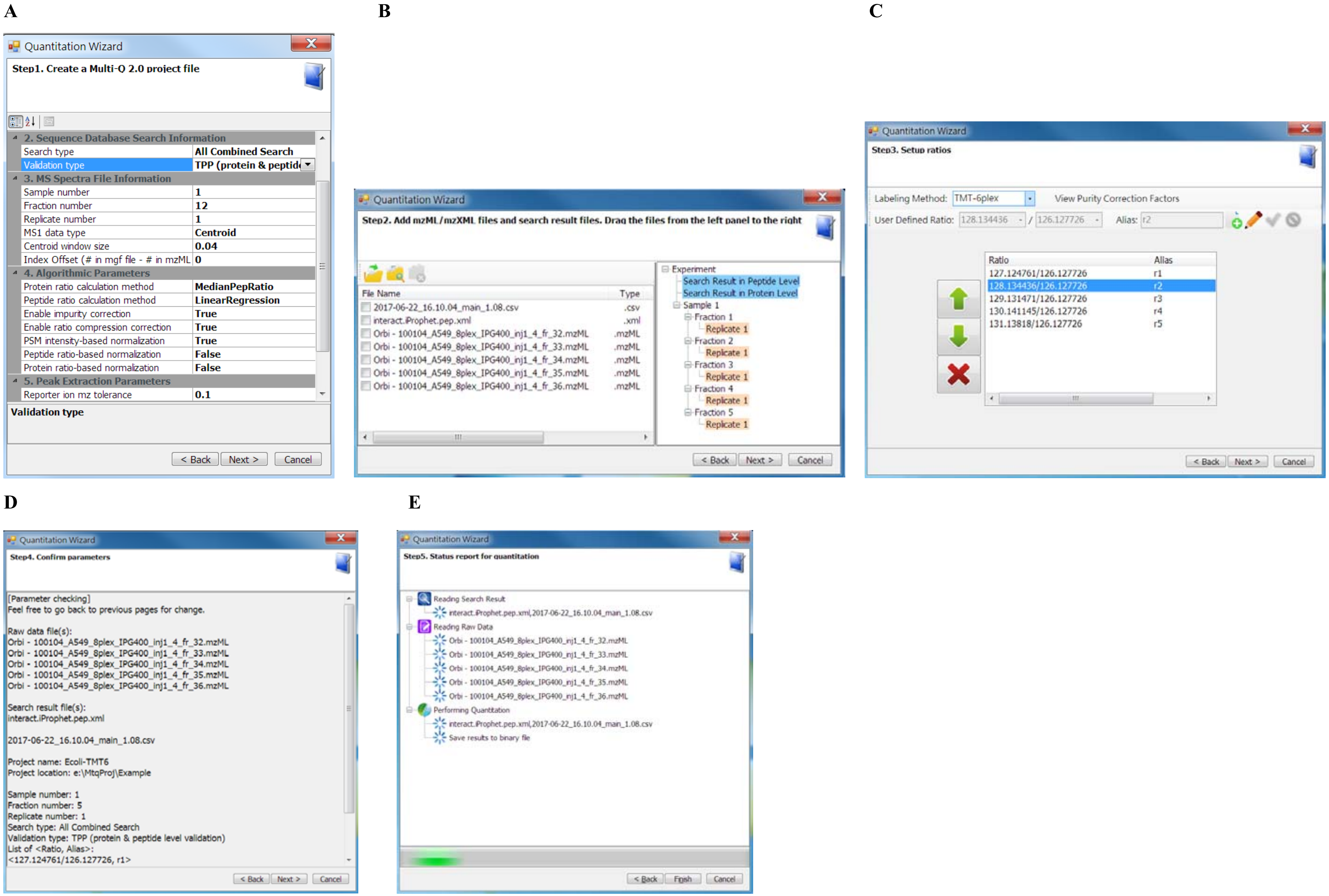
A
Quantitation Wizard
Step1. Create a Multi-Q 2.0 project file
2. Sequence Database Search Information
Search type
All Combined Search
Validation type
TPP (protein & peptid
3. MS Spectra File Information
Sample number
1
Fraction number
12
Replicate number
1
MS1 data type
Centroid
Centroid window size
0.04
Index Offset (# in mgf file - # in mzML
0
4. Algorithmic Parameters
Protein ratio calculation method
MedianPepRatio
Peptide ratio calculation method
LinearRegression
Enable impurity correction
True
Enable ratio compression correction
True
PSM intensity-based normalization
True
Peptide ratio-based normalization
False
Protein ratio-based normalization
False
5. Peak Extraction Parameters
Reporter ion mz tolerance
0.1
Validation type
< Back
Next >
Cancel
B
Quantitation Wizard
Step2. Add mzML/mzXML files and search result files. Drag the files from the left panel to the right
File Name
Type
2017-06-22_16.10.04_main_1.08.csv
.csv
interact.iProphet.pep.xml
.xml
Orbi - 100104_A549_8plex_IPG400_inj1_4_fr_32.mzML
.mzML
Orbi - 100104_A549_8plex_IPG400_inj1_4_fr_33.mzML
.mzML
Orbi - 100104_A549_8plex_IPG400_inj1_4_fr_34.mzML
.mzML
Orbi - 100104_A549_8plex_IPG400_inj1_4_fr_35.mzML
.mzML
Orbi - 100104_A549_8plex_IPG400_inj1_4_fr_36.mzML
.mzML
Experiment
Search Result in Peptide Level
Search Result in Protein Level
Sample 1
Fraction 1
Replicate 1
Fraction 2
Replicate 1
Fraction 3
Replicate 1
Fraction 4
Replicate 1
Fraction 5
Replicate 1
< Back
Next >
Cancel
C
Quantitation Wizard
Step3. Setup ratios
Labeling Method:
TMT-6plex
View Purity Correction Factors
User Defined Ratio:
128.134436
126.127726
Alias:
r2
Ratio
Alias
127.124761/126.127726
r1
128.134436/126.127726
r2
129.131471/126.127726
r3
130.141145/126.127726
r4
131.13818/126.127726
r5
< Back
Next >
Cancel
D
Quantitation Wizard
Step4. Confirm parameters
[Parameter checking]
Feel free to go back to previous pages for change.
Raw data file(s):
Orbi - 100104_A549_8plex_IPG400_inj1_4_fr_32.mzML
Orbi - 100104_A549_8plex_IPG400_inj1_4_fr_33.mzML
Orbi - 100104_A549_8plex_IPG400_inj1_4_fr_34.mzML
Orbi - 100104_A549_8plex_IPG400_inj1_4_fr_35.mzML
Orbi - 100104_A549_8plex_IPG400_inj1_4_fr_36.mzML
Search result file(s):
interact.iProphet.pep.xml
2017-06-22_16.10.04_main_1.08.csv
Project name: Ecoli-TMT6
Project location: e:\MtqProj\Example
Sample number: 1
Fraction number: 5
Replicate number: 1
Search type: All Combined Search
Validation type: TPP (protein & peptide level validation)
List of <Ratio, Alias>:
<127.124761/126.127726, r1>
< Back
Next >
Cancel
E
Quantitation Wizard
Step5. Status report for quantitation
Reading Search Result
interact.iProphet.pep.xml,2017-06-22_16.10.04_main_1.08.csv
Reading Raw Data
Orbi - 100104_A549_8plex_IPG400_inj1_4_fr_32.mzML
Orbi - 100104_A549_8plex_IPG400_inj1_4_fr_33.mzML
Orbi - 100104_A549_8plex_IPG400_inj1_4_fr_34.mzML
Orbi - 100104_A549_8plex_IPG400_inj1_4_fr_35.mzML
Orbi - 100104_A549_8plex_IPG400_inj1_4_fr_36.mzML
Performing Quantitation
interact.iProphet.pep.xml,2017-06-22_16.10.04_main_1.08.csv
Save results to binary file
< Back
Finish
Cancel

Figure S10. Screenshots of Multi-Q 2 quantitation wizard.

(A) Parameter settings regarding quantitation algorithms, (B) selection of input files, (C) specifying labeling method and ratios, (D) confirmation of all the parameters, and (E) progress for quantitation. Details of the wizard are available in the user manual on the website of Multi-Q 2.

Figure S11.

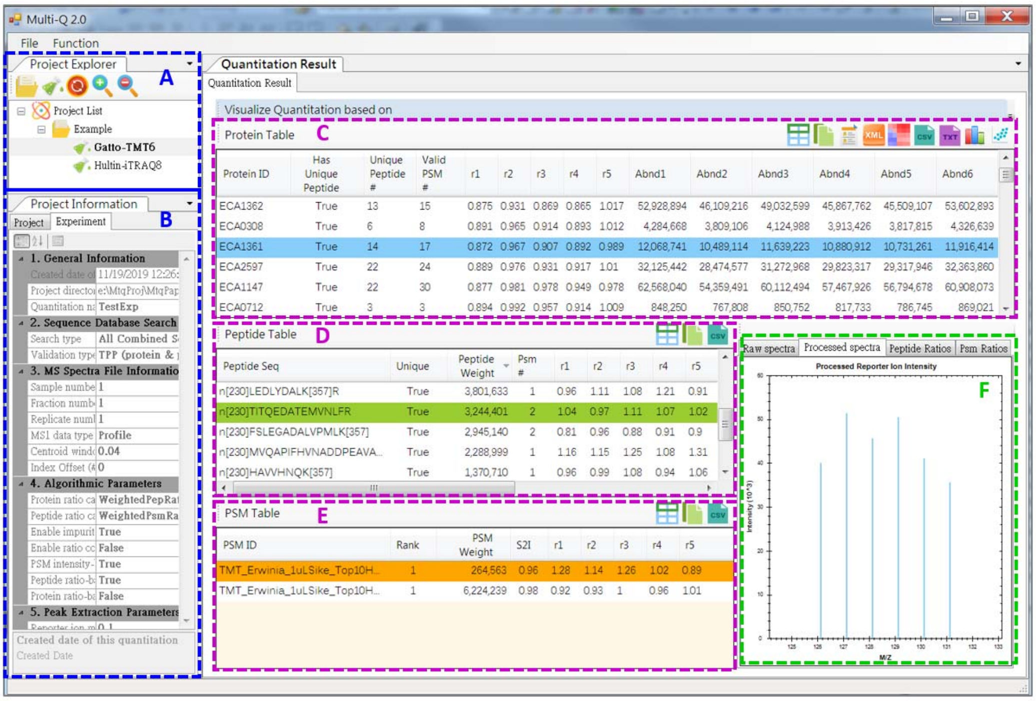

Figure S11. Main window of Multi-Q 2.

Project explorer (panel A) shows the identifiers of isobaric labeling experiments. Project information (panel B) shows all the parameters for quantitation. Protein table (panel C) shows the summary of protein quantitation results, including protein identifier, number of unique peptides, number of PSMs, protein ratios, and protein-level reporter ion abundances for all the quantified proteins in the data set. Clicking on a protein in the protein table, peptide table (panel D) shows the results of all the quantified peptides belonging to the protein, including peptide sequence, peptide weight, number of PSMs, peptide ratios, and peptide-level reporter ion abundances of all the peptides. Clicking on a peptide in the peptide table, PSM table (panel E) shows PSM identifier, PSM weight, S2I, and PSM ratios for all the PSMs belonging to the peptide. Visualization panel (panel F) consists of a set of figures in different tabs, including reporter ions from the raw spectra, reporter ions after a series of processing (impurity correction, RCC, and normalization), and distributions of peptide and PSM ratios.

Figure S12.

A

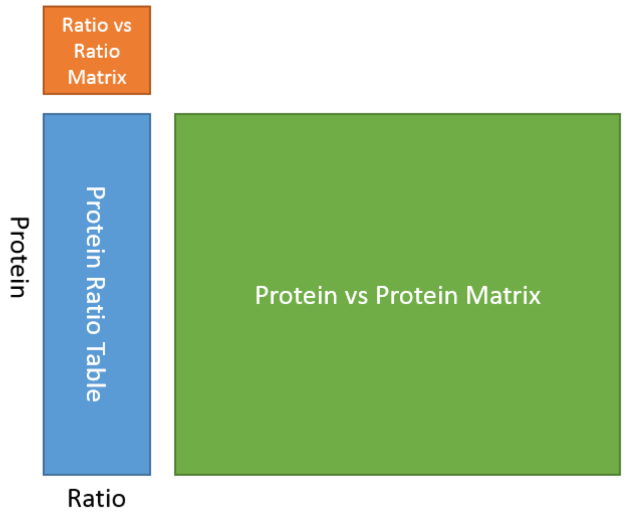

B

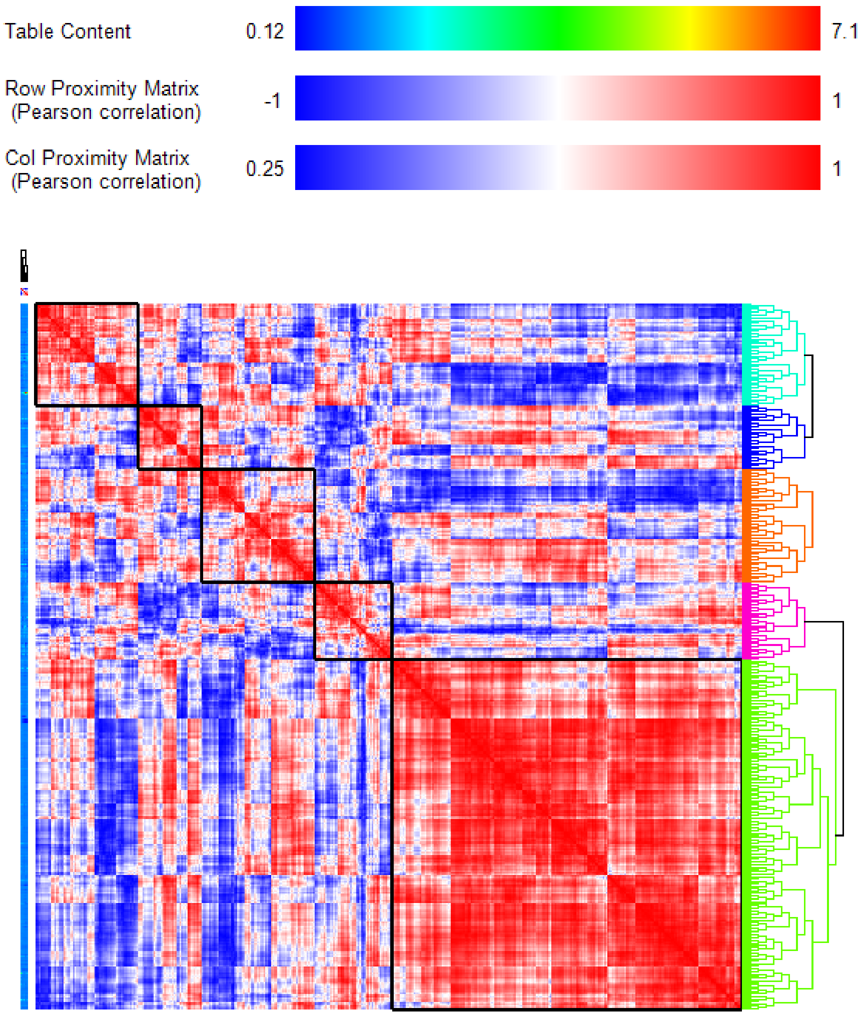

C

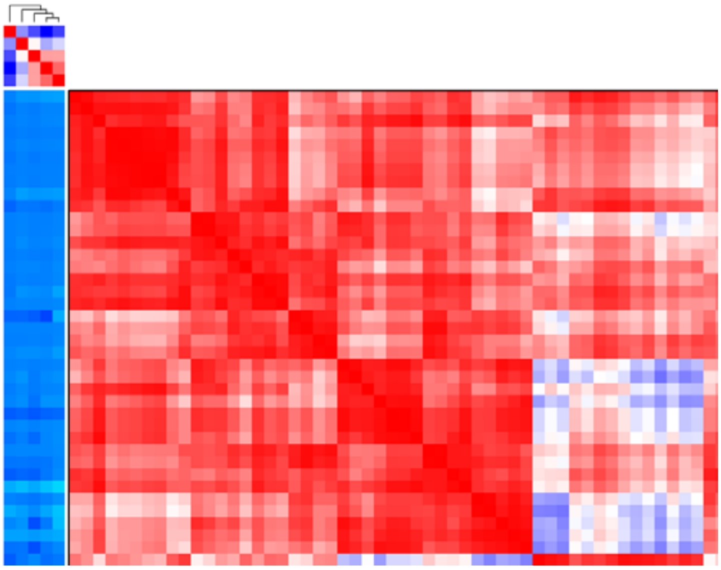

Figure S12. Heatmap module in Multi-Q 2.

(A) Heatmap module consists of three components: a protein ratio table (blue box), a ratio vs. ratio matrix (orange box), and a protein vs. protein matrix (green box). Each component is associated with an individual heatmap. (B) Three color bars from top to bottom correspond to protein ratio table, protein vs. protein matrix, and ratio vs. ratio matrix, respectively. Five clusters are displayed in the protein vs. protein heatmap. (C) A zoomed-in image from the upper left corner of the entire heatmap shows the ratio vs. ratio matrix, a dendrogram, a partial protein ratio table, and a partial protein vs. protein matrix.

Figure S13.

A

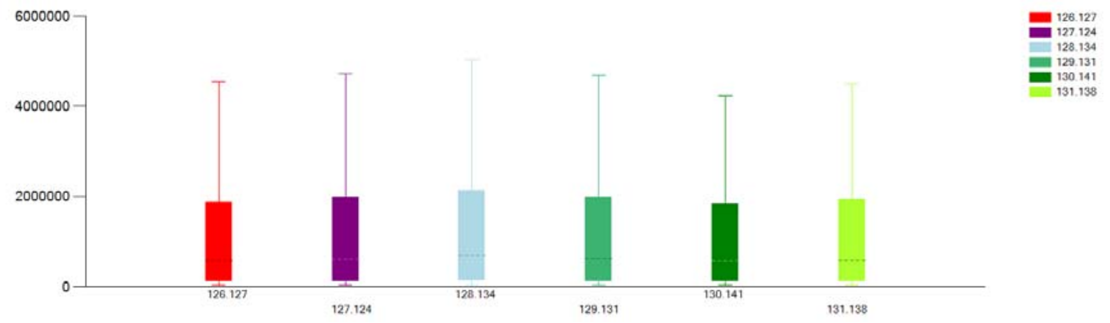

B

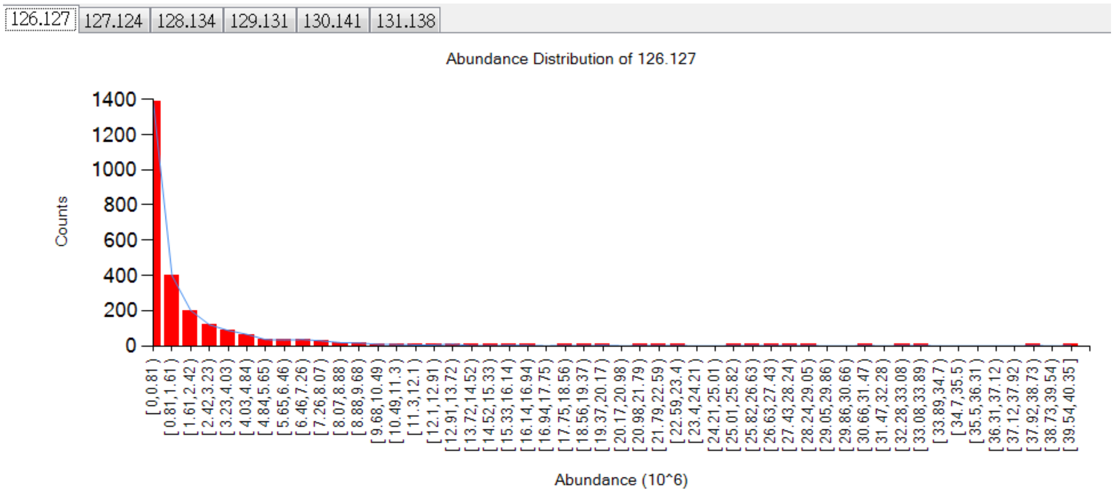

Figure S13. Screenshots of Multi-Q 2 regarding distributions of reporter ion intensities.

(A) Boxplots of reporter ion intensities for six channels in a TMT6 experiment. (B) A bar chart of abundance distributions of channel 126.127 in a TMT6 experiment.

Figure S14.

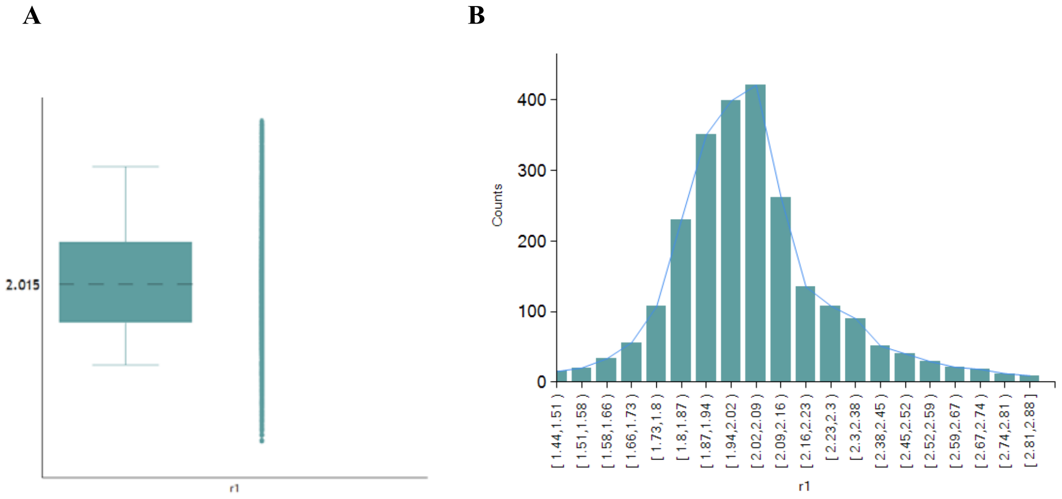

Figure S14. Screenshots of Multi-Q 2 regarding distributions of protein ratios.

(A) A boxplot of protein ratios in a TMT6 experiment. (B) A bar chart of the distribution of protein ratios in a TMT6 experiment.
